# Measurement of Flow Fluctuation in the Flow Standard Facility Based on Singular Value Decomposition

**DOI:** 10.3390/s21206850

**Published:** 2021-10-15

**Authors:** Tao Meng, Huanchang Wei, Feng Gao, Huichao Shi

**Affiliations:** 1 Division of Thermophysics Metrology, National Institute of Metrology, Beijing 100029, China; mengt@nim.ac.cn (T.M.); whc980105@163.com (H.W.); gaof@nim.ac.cn (F.G.); 2 College of Information Science and Technology, Beijing University of Chemical Technology, Beijing 100029, China

**Keywords:** flow standard facility, flow stability, flow fluctuation, singular value decomposition

## Abstract

In order to accurately evaluate the flow stability of the flow standard facility, the flow fluctuation in the standard facility needs to be accurately measured. However, the flow fluctuation signal is always superimposed with the fluctuation signal of the measuring flowmeter or measurement system (mainly noise), which leads to inaccurate measurement of the flow fluctuation and even an unreliable evaluation result of the flow stability. In addition, when there are multiple fluctuation sources, flow fluctuations with different frequencies are superimposed together, which is extremely unfavorable for evaluating the impact of flow fluctuation with different single frequencies. In this paper, a new measuring method was proposed to obtain the fluctuation signal and the flow fluctuation based on singular value decomposition (SVD). Simulation experiments on the fluctuation signal (single frequency and multiple frequencies) under different levels of noise were conducted, and simulation results showed that the proposed method could accurately obtain the fluctuation signal and the flow fluctuation, even under high noise. Finally, an experimental platform was set-up based on a water flow standard facility and a flow fluctuation generator, and experiments on the output signal of a venturi flowmeter were carried out. The experiment results showed that the proposed method could effectively obtain the fluctuation signal and accurately measure the flow fluctuation.

## 1. Introduction

The flow standard facility is a key device for providing accurate and stable flow values for calibrating and evaluating the performance of flowmeters [1]. The flow accuracy of the flow standard facility is estimated with uncertainty, and flow stability refers to the fluctuation degree of the flow with time [2]. With the decrease in uncertainty of the flow standard facility, the flow stability has been gaining increasing attention [3,4]. Flow stability not only affects the flow accuracy of the water flow standard facility [5,6,7], but also brings large uncertainty to the calibration results [8,9,10]. In order to improve the flow stability of the flow standard facility, it is necessary to measure the flow fluctuation accurately.

A flow fluctuation in the flow standard facility may be generated by rotary or reciprocating positive displacement engines, compressors, and pumps, and the fluctuation signal in a general flow standard facility could be regarded as a sinusoidal signal [8,11]. Due to the various types of fluctuation and their complex mechanisms, it is difficult to measure the flow fluctuation directly and accurately [8]. Existing flow stability measurement methods generally use a single flowmeter, and the standard deviation of the measured results is used to characterize the flow fluctuation [5].

However, in addition to the possible distortion of measurement results due to the unstable performance of the flowmeter, it is difficult to distinguish the flow fluctuation from noise, pipeline vibration, and electrical signal interference [12]. In particular, the fluctuation signal of a flowmeter or measurement system (mainly noise) is superimposed on the signal of the flow fluctuation, even noise that is stronger than the flow fluctuation signal, which makes the measured result of the flow fluctuation inaccurate. In addition, when there are multiple fluctuation sources, flow fluctuations with different frequencies are superimposed together, which is extremely unfavorable for evaluating the impact of the flow fluctuation with different single frequencies.

The flow fluctuation signal measured from the flow standard facility and the noise of the flowmeter or measurement system are uncertain, and noise is usually regarded as white noise [5]. Noise reduction methods, such as the narrowband filter and time-domain averaging method, could be used; however, noise cannot be completely filtered out and the flow fluctuation signal may be attenuated [13]. Other available filtering methods, such as the median filter [14], frequency and wavenumber domain filtering (f-k filtering) [15], and wavelet transform method [16], also have shortcomings in eliminating noise in the flow fluctuation signal. The median filter and f-k filtering could eliminate noise to some extent, but may cause loss to the main signal too. The wavelet transform method is based on wavelet decomposition, and it could eliminate random noise and ensure that the useful information of the main signal is not lost to the greatest extent. However, it is difficult to select the wavelet base that has the greatest influence on the result of noise reduction, and the effect of noise reduction is poor for the white noise.

Therefore, a new method that can effectively separate noise from the flow fluctuation signal without causing energy loss of the flow fluctuation signal itself is needed, and it should be able to extract the flow fluctuation with a single frequency for the situation where flow fluctuations with different frequencies are superimposed together. Singular value decomposition (SVD) could divide the noisy signal vector space into different subspaces that are dominated by pure signal and noise, and then the pure signal could be obtained by removing the vector components that fall in the noise subspaces [17,18,19]. On this basis, a single-frequency signal could be extracted separately from the superposition signal of multiple flow fluctuations according to the singular values and its corresponding vectors. This method provides a possible way to obtain the flow fluctuation signal and the flow fluctuation accurately.

In this paper, a new measuring method based on SVD was proposed to obtain the fluctuation signal and the flow fluctuation. Data reconstruction was carried out to expand single-dimensional data of the measured result of the flowmeter to multi-dimensional data by the Hankel matrix construction method, in order to realize noise reduction of the flow fluctuation signal and extraction of the fluctuation signal with a single frequency. Simulation experiments on different fluctuation signals under different levels of noise were conducted, after influences of the data selection window width and number of singular values on measured results were analyzed. Finally, an experimental platform was set-up based on a water flow standard facility and a flow fluctuation generator, and experiments on the output signal of a venturi flowmeter were carried out by the proposed method.

## 2. Flow Stability of the Flow Standard Facility

The flow stability of the flow standard facility refers to the ability of the metering performance of the facility to remain constant when the working conditions are constant. The flow stability not only affects the accuracy and repeatability of the measured results of the flowmeter, but also affects the unity and accuracy of the flow value transmission and traceability [20,21]. In order to improve the flow stability of the flow standard facility and evaluate the calibration result of the flowmeter, it is necessary to obtain the quantitative index of the flow stability accurately. The flow fluctuation is the most concerned quantitative index, and it is expressed by the relative standard deviation of the flow rate measured by a single flowmeter in a period of time [22]. In addition, the flow fluctuation is

(1) $$D_{q}=\frac{\sigma }{\mu }=\frac{\sqrt{\frac{1}{N}\cdot {\sum_{i=1}^{N}\left(q_{i}-\mu \right)}^{2}}}{\frac{1}{N}\cdot \sum_{i=1}^{N}q_{i}}$$

where N is the measurement times, $q_{i}$ is the flow rate measured for the *i*-th time, and $i=1,2\ldots \ldots N$. σ is the standard deviation of the measured results over a period of time, and μ is the average flow rate of N measurements. The fluctuation signal in the general flow standard facility can be regarded as a sinusoidal signal [8,11]. When the flow fluctuation signal is a sinusoidal signal with a single frequency, the power of the signal is the variance of the measured results, and

(2) $$\sigma^{2}=\frac{A^{2}}{2}$$

where *A* is the amplitude of the sinusoidal signal, and the flow fluctuation can be obtained by directly measuring the amplitude of the sinusoidal signal. However, there are a lot of noise in the measured flow fluctuation signal, and flow fluctuation signals are superimposed when there are multiple fluctuation sources. It is difficult to directly obtain the amplitude of the sinusoidal flow fluctuation signal to evaluate the flow stability of the flow standard facility.

## 3. Principle of Proposed Measuring Method Based on SVD

The measuring method of the flow fluctuation proposed in this paper is based on SVD, and it is called SVD method for short. Through SVD, the signal measured by the flowmeter can be divided into several subspaces that represent different signals, such as flow fluctuation signals with different frequencies and noise. After selecting different subspaces, the signal can be recovered using the singular values and their corresponding vectors, and then the flow fluctuation signal can be obtained.

For the flow fluctuation signal with noise, the noisy signal vector space can be decomposed into two spaces. One is dominated by the pure signal, and the other is dominated by noise. Then, the noise vector falling in the noise space is removed to estimate the pure signal. Assume that the matrix M is a data matrix of the measured signal with noise:

(3) M=S+N

where S is the signal data matrix containing the pure signal, N is the noise data matrix, and all three matrices are *n* × *m* matrices. For the signal matrix S, its rank $R(S)=r_{S}<m$, and it can be decomposed by SVD as follows:

(4) $$S=U_{x}\Sigma_{x}V_{x}=(U_{x1},U_{x2})\left[\begin{array}{cc} \Sigma_{x1} & 0\\ 0 & 0 \end{array}\right]\left[\begin{array}{c} V_{x1}\\ V_{x2} \end{array}\right]$$

where $U_{x1}\in S_{n\times r},U_{x2}\in S_{n\times (n-r)},V_{x1}\in S_{r\times r},V_{x2}\in S_{(n-r)\times m}$ are left or right singular vectors of S, and $\Sigma_{x}$ is a diagonal matrix of the singular values $\sigma_{k}(k=1,2,\cdots ,r_{S})$ of signal matrix S.

According to the properties of a unitary matrix:

(5) $$\left\{ \begin{array}{l} V_{x1}V_{x1}^{T}=I\\ V_{x2}V_{x2}^{T}=I\\ V_{x1}V_{x1}^{T}+V_{x2}V_{x2}^{T}=I \end{array}\right.$$

A mixed matrix M with noise and signal can be decomposed by SVD:

(6) $$\begin{array}{l} M=S+N\\ =SV_{x1}V_{x1}^{T}+N(V_{x1}V_{x1}^{T}+V_{x2}V_{x2}^{T})\\ =(SV_{x1}+NV_{x1})V_{x1}^{T}+(NV_{x2})V_{x2}^{T}\\ =(P_{1}\Lambda_{1}Q_{1}^{T})V_{x1}^{T}+(P_{2}\Lambda_{2}Q_{2}^{T})V_{x2}^{T}\\ =\left(P_{1},P_{2}\right)\left[\begin{array}{cc} \Lambda_{1} & 0\\ 0 & \Lambda_{2} \end{array}\right]\left[\begin{array}{c} Q_{1}^{T}V_{x1}^{T}\\ Q_{2}^{T}V_{x2}^{T} \end{array}\right] \end{array}$$

where $P_{1}$ and $P_{2}$ are left singular vectors of M, and $Q_{1}^{T}V_{x1}^{T}$ and $Q_{2}^{T}V_{x2}^{T}$ are right singular vectors of M. For efficient SVD, $P_{1}^{T}P_{2}=0$, which means that vector $P_{1}$ and vector $P_{2}$ are orthogonal. Multiple singular values could be obtained by decomposition and form diagonal matrices $\Lambda_{1}$ and $\Lambda_{2}$ respectively.

Among these singular values, vectors corresponding to relatively large singular values contain more information, while vectors corresponding to smaller singular values contain less information. In this case, it could be considered that vectors corresponding to relatively large singular values are vectors in signal subspace, while vectors corresponding to smaller singular values are vectors in noise subspace. In addition, the pure signal in the noisy signal could be obtained by inverse calculation after smaller singular values and its corresponding vectors are deleted. After SVD of M, $P_{1}\neq U_{x1}$, signal matrix S could not be obtained directly. By using the least squares method, the estimation of signal matrix $S^{\prime }$ with the least squares error can be obtained [19].

(7) $$S^{\prime }=\sum_{k=1}^{r}\sigma_{k}U_{k}V_{k}^{T}$$

where $\sigma_{k}(k=1,2,\cdots ,r,\;r<r_{S})$ are reserved r singular values after deleting small singular values and $\sigma_{1}>\sigma_{2}>\cdots >\sigma_{r}$. When the flow fluctuation signal is composed of multiple-frequency flow fluctuations, the singular values could be grouped according to their values, and corresponding vectors of these singular values could be combined to recover the flow fluctuation signal with different single frequencies. In this way, the single-frequency flow fluctuation signal could be separated from the superimposed fluctuation signal.

The measuring method of the flow fluctuation proposed in this paper could eliminate noise in the measured results of the flowmeter or measurement system and accurately obtain the real flow fluctuation signal from the noisy signal or superposition flow fluctuation signal of multiple frequencies, and then obtain an accurate flow fluctuation in the flow standard facility.

According to principle of the measuring method of flow fluctuation, data reconstruction should be carried out first to expand single-dimensional data of the measured results to multi-dimensional data [23]. The Hankel matrix construction method [23] was used here. Reconstruction of the single-dimensional data with the total length of *N*: select the first *m* data in the single-dimensional dataset as the first row of the new matrix; the second data to the (*m* + 1)-th data as the second row; the third data to the (*m* + 2)-th data as the third row; and so on, until the *n*-th data to (*m* + *n* − 1)-th data as the last row. The new matrix after reconstruction is

(8) $$X=\left[\begin{array}{l} x_{1},x_{2},x_{3},\cdots ,x_{m}\\ x_{2},x_{3},x_{4},\cdots ,x_{m+1}\\ x_{3},x_{4},x_{5},\cdots ,x_{m+2}\\ \cdots \cdots \\ x_{n}{,}_{}x_{n+1}{,}_{}x_{n+2},\cdots ,x_{m+n-1} \end{array}\right]$$

where *m* is the width of the data selection window. After data reconstruction, an *n* × *m* multi-dimensional data matrix is formed, in which each column represents a variable and each row represents a sample.

## 4. Simulation Experiment

### 4.1. Generation and Pre-Processing of Simulated Flow Fluctuation Signal

The fluctuation signal in the general flow standard facility could be regarded as a sinusoidal signal [8,11]. In order to verify the effectiveness of the proposed measuring method, two sinusoidal fluctuation signals generated by simulation and their superposition signal were used to represent the flow fluctuation signal obtained from the flow standard facility, respectively. Gaussian random noise with zero mean and different variances represents noise generated by the flowmeter or measurement system. Simulated fluctuation signals and noise were generated in MATLAB (Version R2014a), and the simulation experiment was also carried out in MATLAB.

The frequencies of two sinusoidal fluctuation signals were 1 Hz and 8 Hz, and the flow fluctuations were 1% and 0.5% respectively. The simulated average flow rate was 50 m^3^/h. According to Equations (1) and (2), flow fluctuations 1% and 0.5% correspond to the amplitudes of the sinusoidal fluctuation 0.707 m^3^/h and 0.354 m^3^/h, respectively. For the simulation and experiment, the sampling frequencies were set as 20 Hz and 160 Hz for sinusoidal fluctuation signals of 1 Hz and 8 Hz, respectively, and the sampling frequency was set as 160 Hz when the two signals were superposed. A quantity of 1000 data points were collected continuously as the result data for different fluctuation signals in the simulation experiment.

In order to realize the measuring method based on SVD, data reconstruction was carried out first to expand single-dimensional data to multi-dimensional data by the Hankel matrix construction method [23]. The one-dimensional simulated data of the flow fluctuation signal and later experimental data were reconstructed into a multi-dimensional matrix. The constructed matrix was similar to that in Equation (8), and the total length *N* of the single-dimensional data was 1000. The width of the data selection window was *m*, and *m* + *n* − 1 = 1000.

For the flow fluctuation signal generated by simulation, Gaussian random noise with a mean value of 0 and different variance was loaded. The noise variances were 0.01, 0.1, and 1. According to Equation (1), the flow fluctuation with different magnitudes of noise could be calculated, and the calculated results reflect the influence of noise on the measured result of flow fluctuation. Simulated flow fluctuation signals were also processed by the median filter, and the results were used to compare with the results of the proposed method. A median filter is a kind of nonlinear filtering, which is suitable for cases where noise characteristics are not well known or noise overlaps with the spectrum of the signal.

In order to evaluate the flow fluctuation signals obtained by the proposed method and median filter, the amplitude attenuation and signal-to-noise ratio (SNR) were calculated after the simulated flow fluctuation signal without noise was used to subtract the signals obtained by the proposed method and median filter data-to-data, respectively. In addition, the mean difference of the sinusoidal peak of the simulated flow fluctuation signal without noise and the signal obtained by the proposed method or median filter was used as the amplitude attenuation. The variance of the peak value difference was used to calculate the SNR of the obtained signal.

### 4.2. Parameters Setting of Measuring Method Based on SVD

In order to obtain an accurate flow fluctuation, it is necessary to study influences of the width of the data selection window *m* and number of singular values selected. The optimal window width and number of singular values were selected by comparing the result. Flow fluctuation signals simulated and superposed with random noise with 0 mean and 0.01 variance were used. Frequencies of two fluctuation signals were 1 Hz and 8 Hz, and fluctuation amplitudes were 0.707 m^3^/h and 0.354 m^3^/h, respectively.

**Figure 1:**
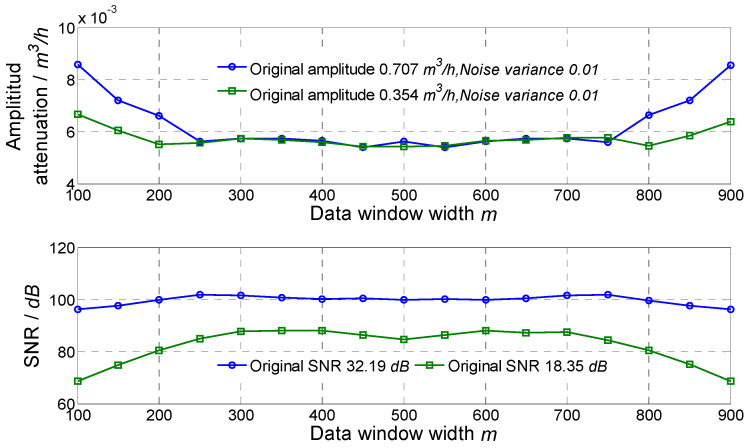
Result of using different data window widths.

For different widths of the data selection window, the amplitude attenuation and SNR of the signal obtained by the proposed method are shown in Figure 1. For a window width between 400 and 600, amplitude attenuations of the obtained sinusoidal signals were relatively small. The minimum amplitude attenuation was about 0.8% for the 1 Hz signal when *m* was 450, and the minimum amplitude attenuation was about 1.5% for the 8 Hz signal when *m* was 450 too. The SNR greatly improved for both signals, and the SNRs were basically greater than 80 dB using a window width between 200 and 800. Comprehensively considering the amplitude attenuation and SNR improvement, the window width could be set as 400–600, and 450 was used in the simulation and experiment.

**Figure 2:**
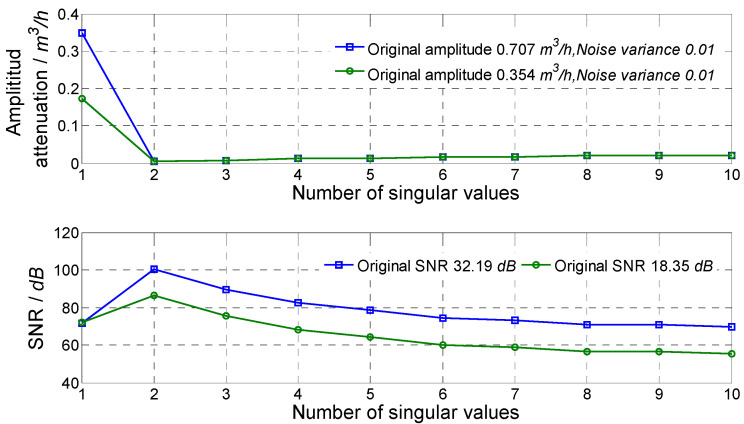
Result of using different numbers of singular values.

For different numbers of singular values, the result of noise reduction is shown in Figure 2. It could be seen that: when one singular value was selected, the amplitude attenuation was relatively large and the improvement of SNR was not good; when two singular values were selected, the amplitude attenuation reduced to a very small value (0.8% for 1 Hz signal and 1.5% for 8 Hz signal), and the SNR improved greatly (greater than 80 dB). Moreover, as the number of selected singular values increased, the SNR decreased, and the amplitude attenuation was slightly larger, which may be caused by noise disturbance. Thus, comprehensively considering amplitude attenuation and SNR improvement, two singular values were used in the simulation and experiment to eliminate noise for the single-frequency flow fluctuation signal.

### 4.3. Simulation Results

In order to verify the effectiveness of the method proposed, two sinusoidal fluctuation signals generated by the simulation and their superposition signal were used to represent the flow fluctuation signal from the flow standard facility. Gaussian random noises with a mean value 0 and noise variances of 0.01, 0.1, and 1 were loaded on the simulated flow fluctuation signal. The simulated flow fluctuation signals were also processed by the median filter, and the results were used to compare with the results of the proposed method.

For the sinusoidal fluctuation signal with a 1 Hz frequency, the flow fluctuation was set as 1%, and the flow fluctuation calculated by Equation (1) increased obviously when noise was added to the simulated flow fluctuation signal. For noise variances of 0.01, 0.1, and 1, the SNR of the simulated flow fluctuation signal were 32.19 dB, 9.16 dB, and −13.86 dB, respectively, and the flow fluctuations were 1.03%, 1.22%, and 2.23%, respectively. It can be seen that noise had a great influence on the measured result. If the noise of the flowmeter or measurement system could not be eliminated, the measured flow fluctuation would contain a great deviation.

**Figure 3:**
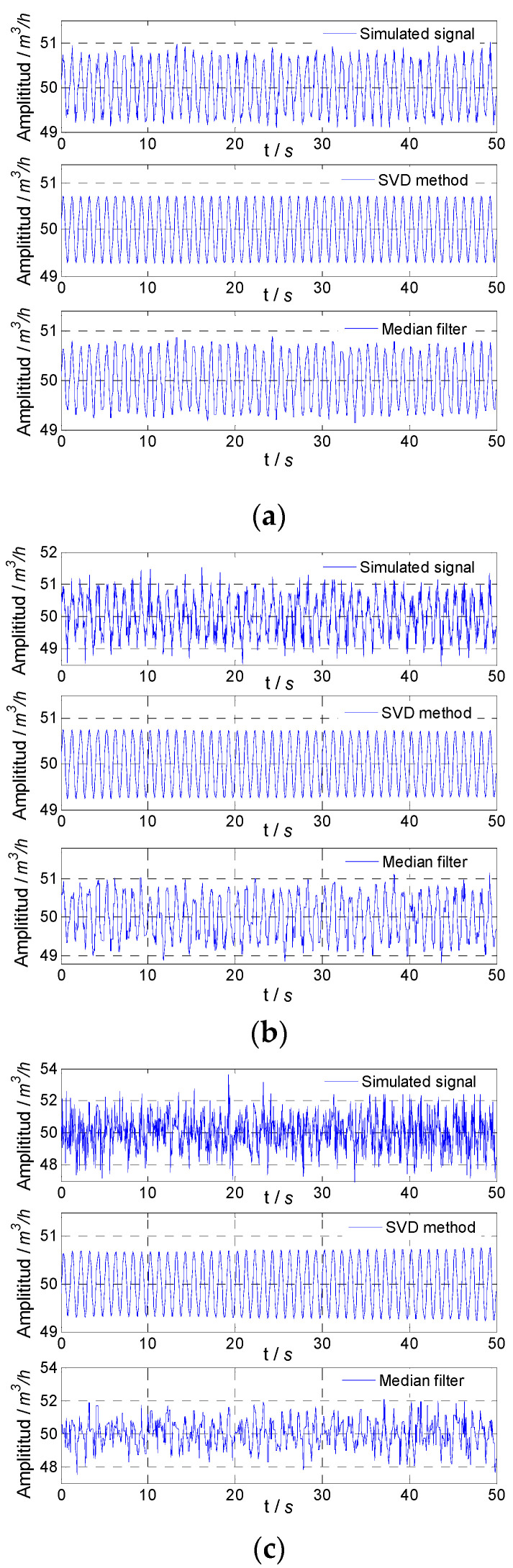
Simulated result for 1 Hz signal: (**a**) noise variance, 0.01; (**b**) noise variance, 0.1; (**c**) noise variance, 1.

The simulated flow fluctuation signals with different noise and signals obtained by the proposed method and median filter are shown in Figure 3. It could be found that with the increase in noise variance, the fluctuation degree of the simulated flow fluctuation signal increased, and the proposed method could accurately obtain the flow fluctuation signal, while the results of the median filter were not good, especially when the noise variance was relatively large.

For the proposed SVD method and median filter, the amplitude attenuation and SNR for the 1 Hz signal are given in Table 1. Comparing the amplitude attenuation of two methods, it could be found that the amplitude attenuation of the proposed method was smaller than that of the median filter. For the proposed method, although amplitude attenuation increased with noise variance, the magnitude of amplitude attenuation had little influence on the calculation results of the flow fluctuation. It could be considered that the proposed method hardly caused energy loss of the simulated flow fluctuation signal.

Comparing the SNR improvement of the two methods, it could be found that the noise reduction effect of the proposed method was better than that of the median filter. For the proposed method, even when the noise variance was 1 (SNR −13.86 dB), the SNR could reach 66.90 dB after noise reduction, which shows that the proposed method had an excellent noise reduction effect and the flow fluctuation signal could be accurately obtained from the noisy signal by this proposed method.

**Figure 4:**
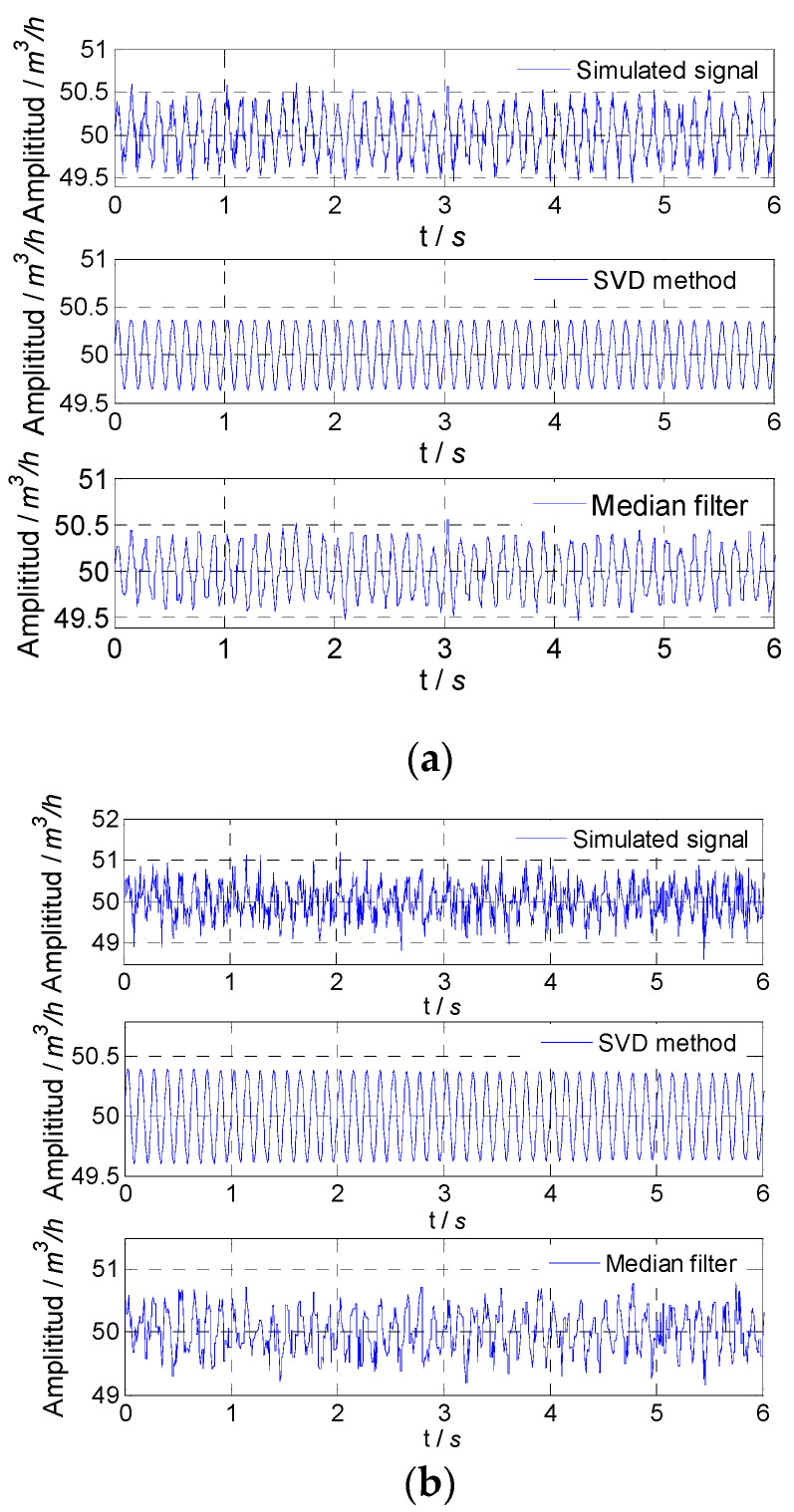

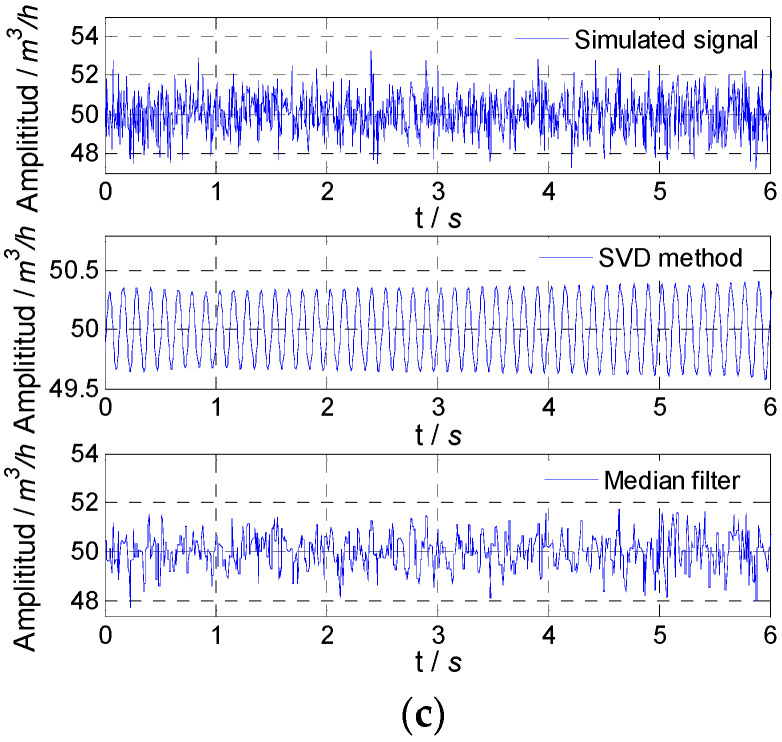
Simulated result for 8 Hz signal: (**a**) noise variance, 0.01; (**b**) noise variance, 0.1; (**c**) noise variance, 1.

For the sinusoidal fluctuation signal with a frequency of 8 Hz, the flow fluctuation was set as 0.5%. The simulated flow fluctuation signal with different noise and the signal obtained by the two methods are shown in Figure 4. For the proposed SVD method and median filter, the amplitude attenuation and SNR improvement are given in Table 2. By comparing the amplitude attenuation and SNR improvement, it could be found that the SVD method could accurately obtain the flow fluctuation signal.

For the superposition fluctuation signal with two frequencies (1 Hz and 8 Hz), the fluctuation of the 1 Hz signal was set as 1%, the fluctuation of the 8 Hz signal was set as 0.5%, and the flow fluctuation calculated by Equation (1) was 1.12%. As the flow fluctuation was formed by the superposition of these two signals, the first four singular values could be divided into two groups corresponding to each frequency signal, after singular value decomposition of the noisy signal. In order to eliminate noise, only these four singular values and their corresponding vectors were used to recover the flow fluctuation signal.

**Figure 5:**
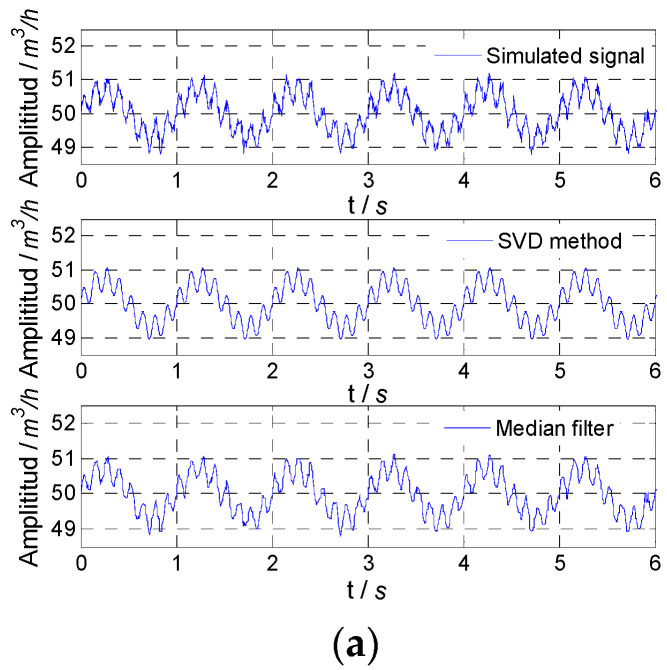

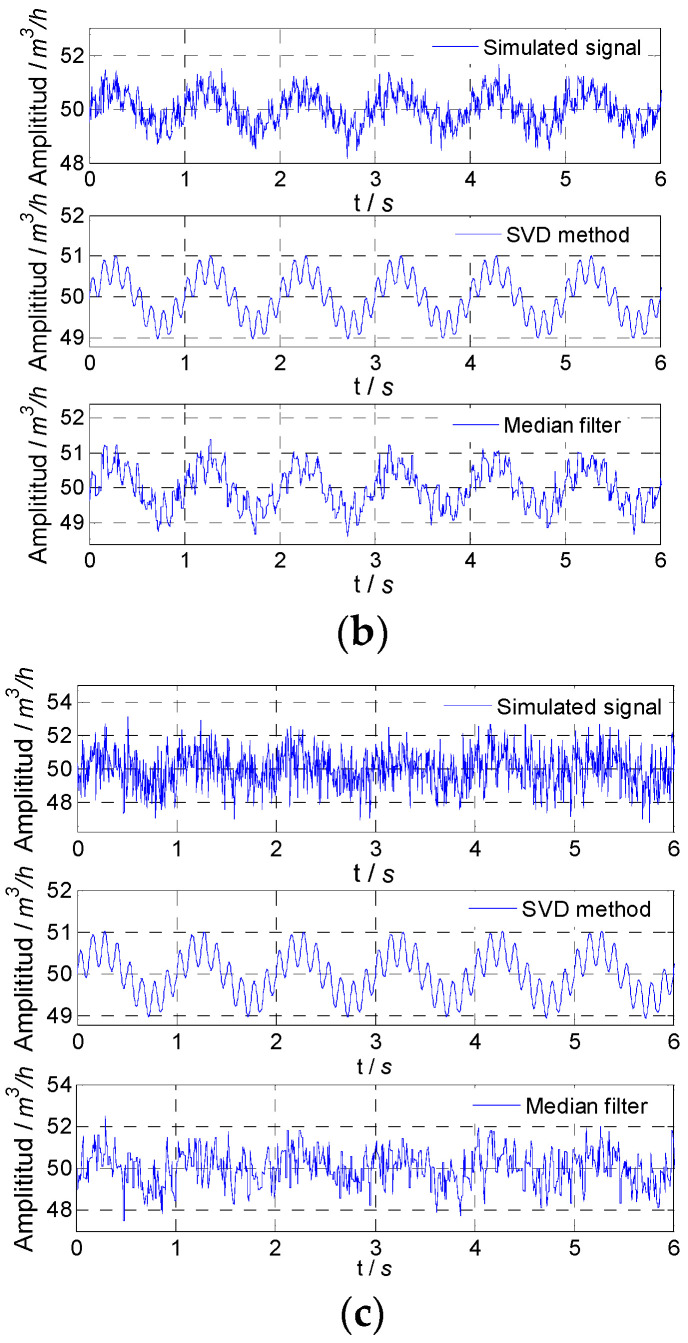
Simulated result for superposition signal: (**a**) noise variance, 0.01; (**b**) noise variance, 0.1; (**c**) noise variance, 1.

The simulated flow fluctuation signal with different noises and signals obtained by the two methods are shown in Figure 5. For the proposed SVD method and median filter, the amplitude attenuation and SNR improvement are given in Table 3. By comparing amplitude attenuation and SNR improvement, it could be found that the SVD method could accurately obtain the superposition fluctuation signal.

**Figure 6:**
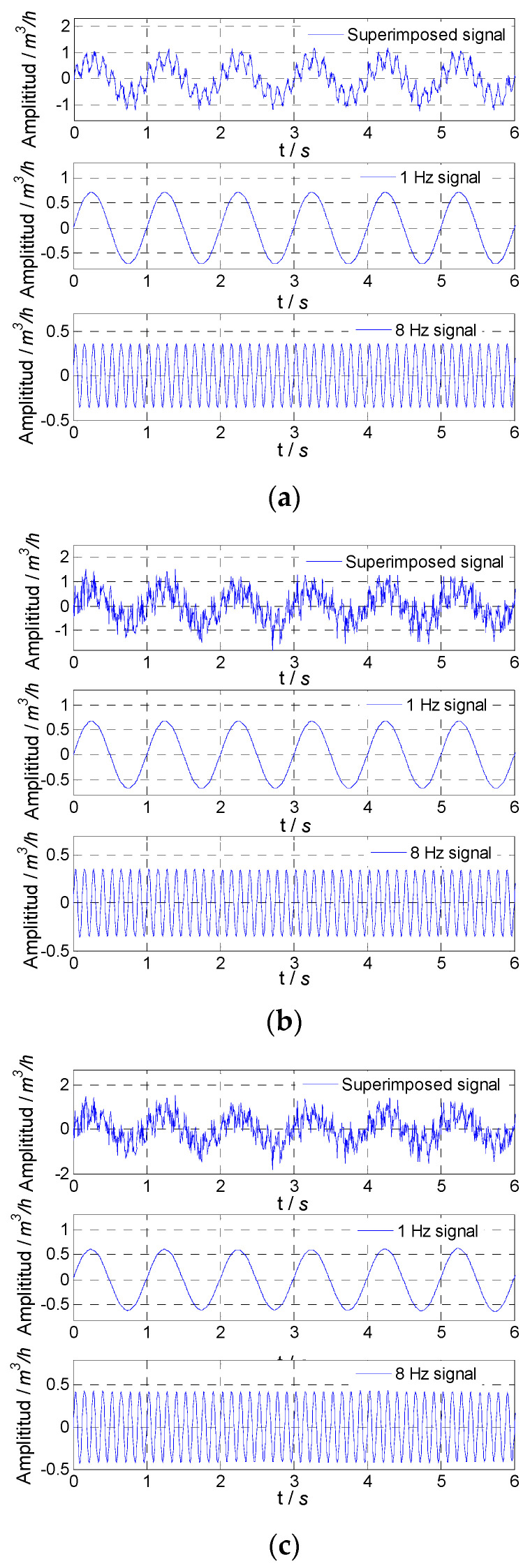
Simulated result for extracted signals: (**a**) noise variance, 0.01; (**b**) noise variance, 0.1; (**c**) noise variance, 1.

For the fluctuation signal formed by superimposing two fluctuation signals with frequencies 1 Hz and 8 Hz, the proposed SVD method could extract two frequency components at the same time of noise reduction. When selecting singular values and corresponding vectors, the first four singular values and corresponding vectors were retained. Among these four singular values, the first two singular values were as one group, the second two singular values were as another group, and the vectors corresponding to each group of singular values were used to recover two frequency signals. The simulated flow fluctuation signals with different noises and extracted flow fluctuation signals with frequencies of 1 Hz and 8 Hz are shown in Figure 6. For the proposed SVD method, amplitude attenuation and SNR improvement are given in Table 4. From Table 4, it could be found that the flow fluctuation signals extracted from the superposition signal had very small amplitude attenuations and very high SNRs. The SVD method could accurately extract the fluctuation signal with different single frequencies from the superposition signal of flow fluctuation signals with different frequencies.

After comparing the amplitude attenuation and SNR improvement of the results obtained by the SVD method and median filter, the measured flow fluctuations by two methods were compared. Flow fluctuations were calculated by Equation (1) using the flow fluctuation signals obtained by the SVD method and median filter. For two flow fluctuations with a single frequency, the simulation results are given in Table 5. It could be found that the results of the SVD method were very close to the real flow fluctuation, and the measured results were less affected by noise with the increase in noise variance, while the results of the median filter and direct calculation worsened with the noise variance. The simulation results showed that the SVD method had good performance in measuring the flow fluctuation.

For the fluctuation signal formed by superimposing two fluctuation signals with frequencies 1 Hz and 8 Hz, the simulation results are given in Table 6. It could be found that the maximum deviation of flow fluctuation was 0.06% when the noise variance was 1, which was far lower than the deviation of direct calculation results and the deviation obtained by the median filter. This shows that the results of the proposed SVD method were very close to the real flow fluctuation than the results of direct calculation and the median filter, and the measured result was less affected by noise with the increase in noise variance. In addition, each frequency fluctuation signal in the superimposed signal could be extracted separately by the SVD method, and the flow fluctuation of each frequency fluctuation could be accurately obtained.

Through analysis and comparison of the simulation results, it could be found that noise had great influence on the directly measured results of the flow fluctuation. If noise was not eliminated, the flow fluctuation obtained might contain large deviations and could not truly reflect the flow stability of the water flow standard facility. The proposed SVD method could accurately obtain the flow fluctuation signal from the noisy signal output by the flowmeter or measurement system, and the obtained flow fluctuation signal had very small amplitude attenuation and high SNR. The proposed SVD method could accurately extract different single-frequency fluctuation signals from the superposition flow fluctuation signal with multiple frequencies. The results of the flow fluctuation calculated using the fluctuation signals obtained by the SVD method were very close to the real flow fluctuations, and the deviations in flow fluctuation caused by noise were very small. In addition, with the increase in the noise variance, the SVD method could still accurately obtain the flow fluctuation without being affected by increased noise variance, compared with the results of direct calculation and the median filter. This shows that the SVD method had an advantage when the SNR of the flow fluctuation signal worsened.

## 5. Experiment and Result

### 5.1. Experiment Setup

**Figure 7:**
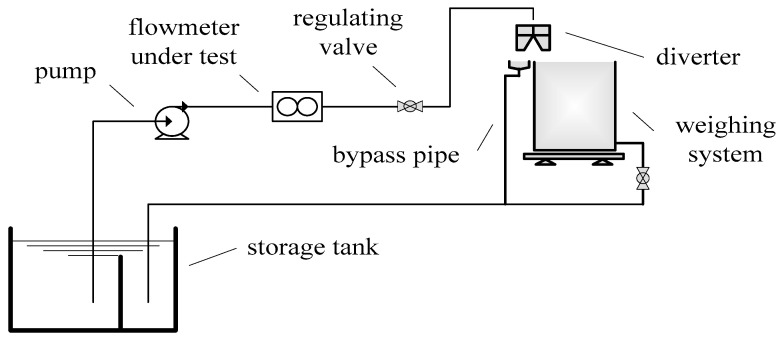
Schematic diagram of the water flow facility.

In order to experimentally verify the effectiveness of the proposed SVD method, an experimental platform was set-up based on a water flow standard facility and a special designed flow fluctuation generator, and experiments on the output signal of a venturi flowmeter were carried out. The experiments were carried out on the water flow standard facility that is in the Flow Laboratory of the National Institute of Metrology of China (NIM). The water flow standard facility is mainly composed of a water storage tank, water pump, measuring flowmeter, regulating valve, bypass, diverter, weighing system, and other main equipment [24], and the schematic diagram is shown as Figure 7.

In the water flow standard facility, the flowmeter is installed in the test section of the pipeline. Water as the fluid medium is pumped from the water tank through the pipeline and measuring flowmeter to the weighing tank. When the flowmeter is calibrated, the water circulation system flows water through the flowmeter to the weighing system, and output values of the weighing system and the flowmeter are compared to determine the measuring performance of the flowmeter.

**Figure 8:**
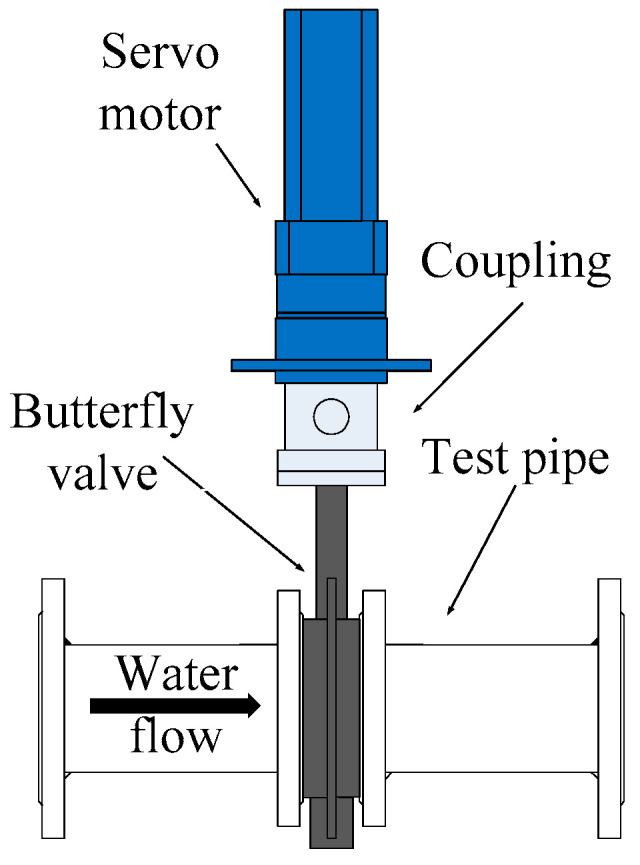
Diagram of the flow fluctuation generator.

After multi-stage flow stabilization, the flow fluctuation in the pipeline is very small. In order to generate controllable flow fluctuation, a flow fluctuation generator that could simulate flow fluctuation was designed, manufactured, and installed at end of the pipeline. The structure of the flow fluctuation generator is shown in Figure 8. The flow fluctuation generator is a valve body of butterfly valves. A servo motor drives the valve to swing around a certain opening degree, and the amplitude of swing could be controlled.

**Figure 9:**
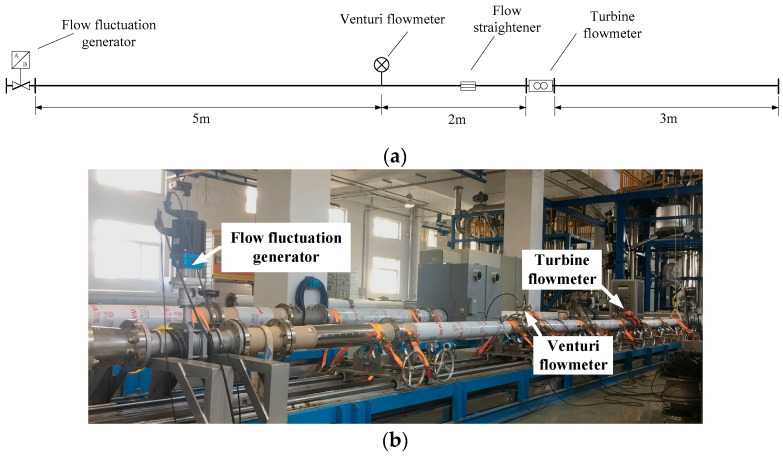
The main pipeline and photo of experimental setup: (**a**) the main pipeline of the flow standard facility; (**b**) photo of experimental setup in the flow standard facility.

Measuring equipment mainly consists of a venturi flowmeter and a flow fluctuation generator in the main pipeline of the flow standard facility, as shown in Figure 9. The main pipeline of the flow standard facility is located on the ground platform in the laboratory, and a turbine flowmeter was installed on the main pipe in order to monitor the flow. The temperature control system of the water flow standard facility was used to keep the water temperature stable, and the temperature of water in the pipeline could be controlled within 10~85 °C.

### 5.2. Experiment Result

In order to verify the effectiveness and correctness of the proposed SVD method, corresponding experiments were carried out on the basis of the simulation experiment. All experimental flow rates were 50 m^3^/h, and the water temperature was 17.2 °C. Due to the limitation of experimental conditions (currently, there is only one flow fluctuation generator), the external flow fluctuation generated by the flow fluctuation generator, the flow fluctuation of the flow standard facility itself in the pipeline, and flowmeter noise were superimposed together and used as the superimposed signal of multi-frequency flow fluctuation signals and noise.

Three experiments were carried out with different experiment conditions. For experiment No. 1, the flow fluctuation generator was not working, the butterfly valve opening was 100%, and the swing range was 0°. At this time, there was no external flow fluctuation, and the measured flow fluctuation came from the flow standard facility itself. For experiment No. 2, the butterfly valve opening was 60%, the valve swinging range was ±5°, and the frequency of the resulting flow fluctuation was 1 Hz. For experiment No. 3, the opening of butterfly valve was 60%, the swing range was ±10°, and the frequency of the resulting flow fluctuation was 8 Hz. For experiment Nos. 2 and 3, in addition to the external flow fluctuation generated by the flow fluctuation generator, there were flow fluctuations in the flow standard facility itself in the pipeline. In the meantime, a certain amount of flowmeter noise was in the measured results, resulting in inaccurate results of the flow fluctuation obtained by direct calculation.

**Figure 10:**
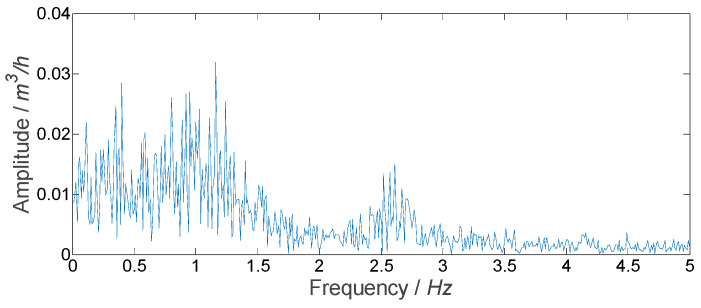
Frequency spectrum of measured result for experiment No. 1.

**Figure 11:**
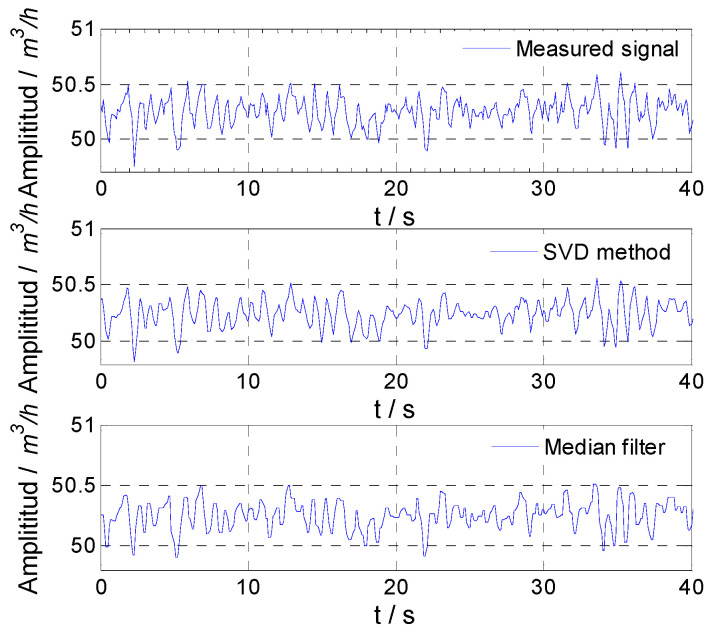
Experimental result for facility fluctuation signal.

For experiment No. 1, the spectrum of the measured result of the flowmeter is shown in Figure 10. It could be seen that the fluctuation of the facility itself had many frequency components that are difficult to separate effectively from the noise. Through singular value decomposition of the flowmeter output, it was found that the singular value corresponding to different frequency components was small and the difference was small too. In order to obtain the flow fluctuation signal from the noisy signal accurately, a proper number of singular values and their corresponding vectors should be selected. The flow fluctuation calculated directly using the measured signal of the flowmeter was 0.25%, and it was 0.22% calculated using the signal obtained by the median filter. Thus, 0.22% was used as the real flow fluctuation to determine the number of singular values selected by the proposed SVD method. When the number of singular values was 100, the flow fluctuation calculated using the signal obtained by the SVD method was 0.22%. Therefore, the number of singular values selected for separating the flow fluctuation signal was set as 100. For experiment No. 1, the flow fluctuation signal of the facility itself obtained by the SVD method and the median filter are shown in Figure 11. Noise could be eliminated by both methods.

**Figure 12:**
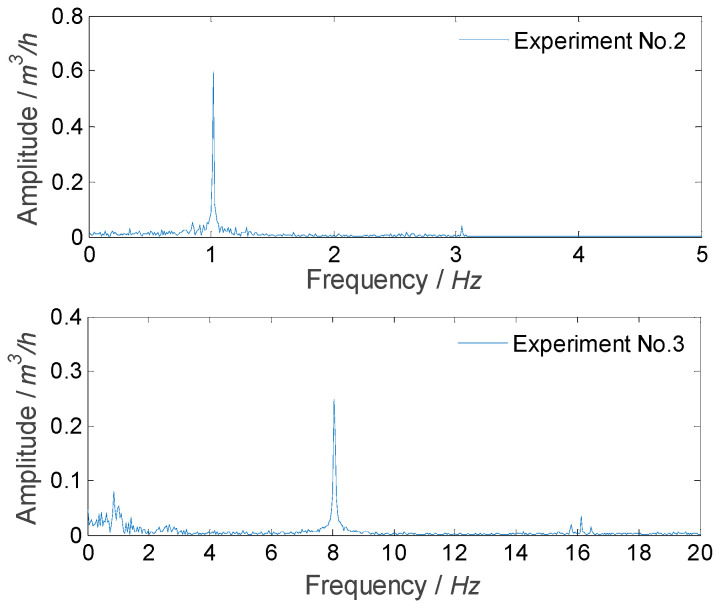
Frequency spectrums of measured result for experiment Nos. 2 and 3.

For experiment Nos. 2 and 3, the measured signal mainly consisted of three parts: sinusoidal fluctuation with a single frequency (1 Hz or 8 Hz), flow fluctuation of facility itself, and noise, as shown in Figure 12. When using the SVD method to obtain the flow fluctuation signal, the first two singular values and their corresponding vectors were selected to extract the sinusoidal fluctuation signal, and then the first 100 singular values in the remaining singular values and their corresponding vectors were selected to extract the fluctuation signal of the facility itself, and other singular values and their corresponding vectors that correspond to noise were deleted.

**Figure 13:**
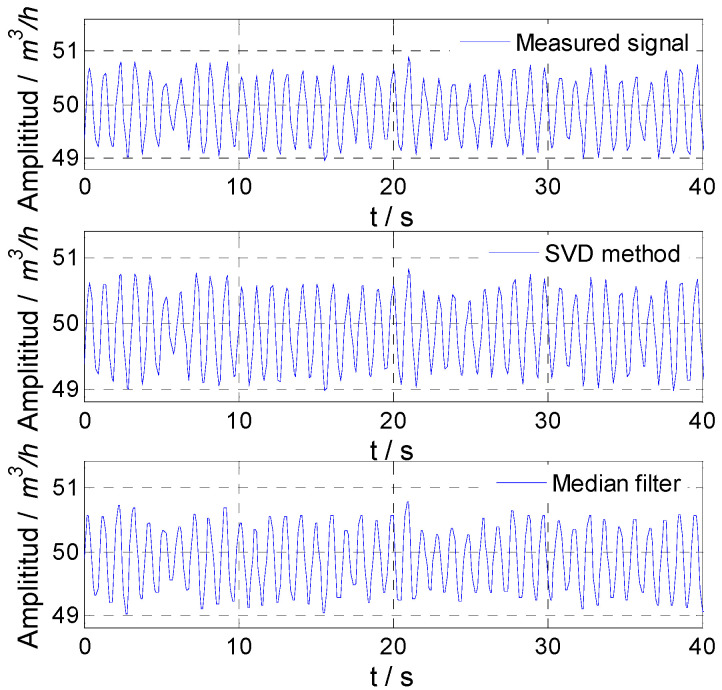
Experimental result for experiment No. 2.

**Figure 14:**
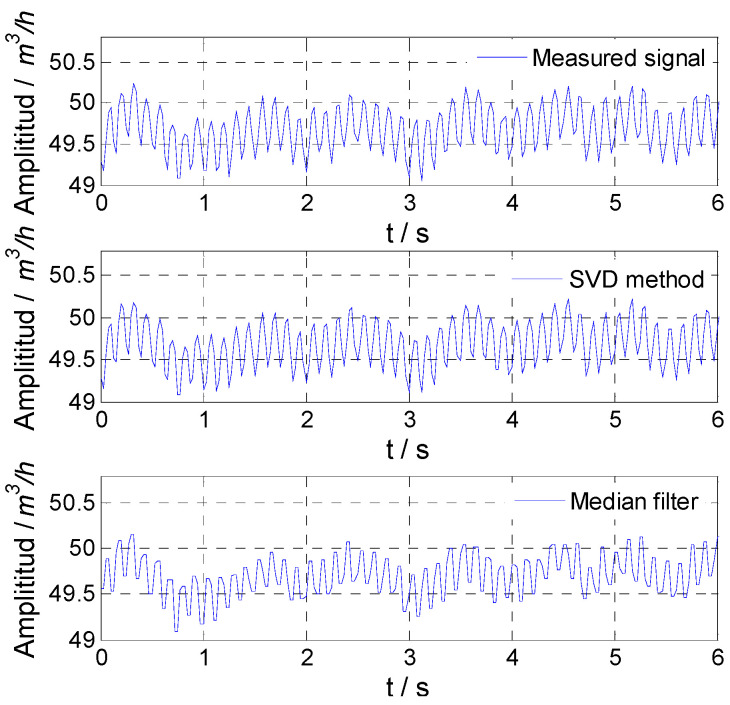
Experimental result for experiment No. 3.

**Figure 15:**
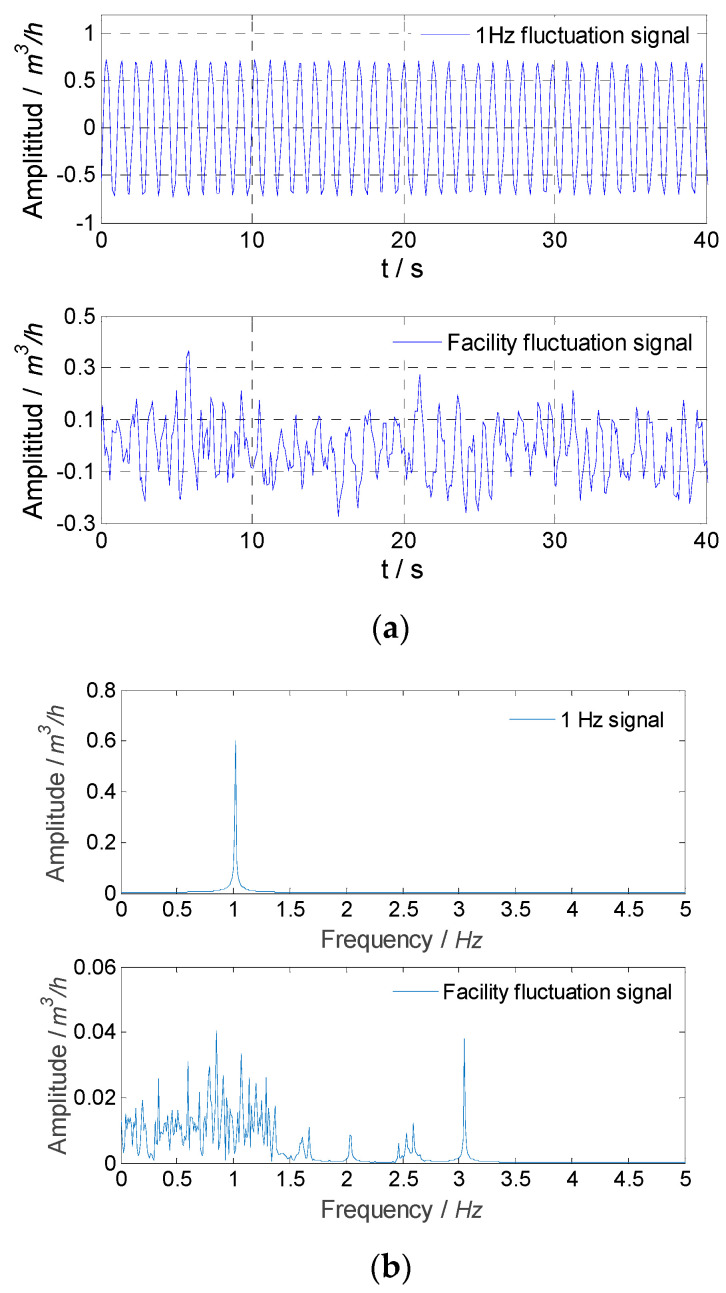
Experimental result for extracted signals in experiment No. 2: (**a**) the flow fluctuation signal; (**b**) frequency spectrums.

**Figure 16:**
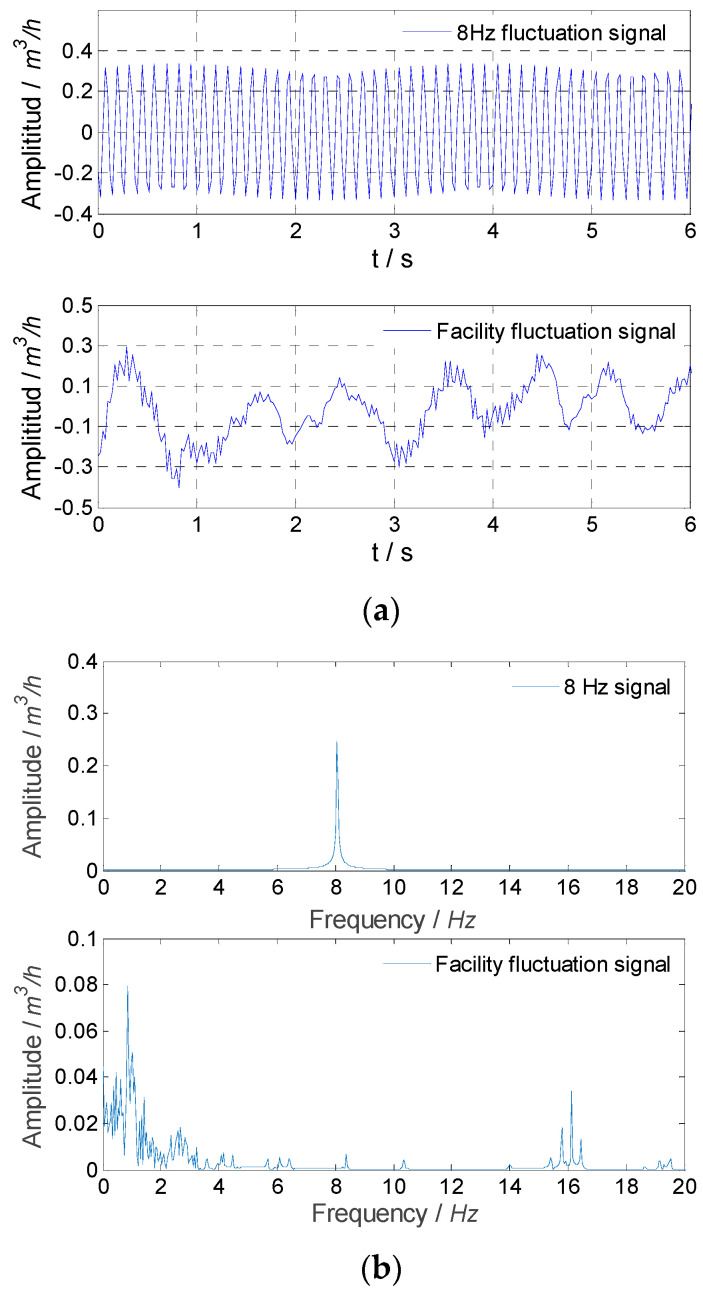
Experimental result for extracted signals in experiment No. 3: (**a**) the flow fluctuation signal; (**b**) frequency spectrums.

For experiment Nos. 2 and 3, the measured signals and signals obtained by the median filter and SVD method are shown in Figure 13 and Figure 14. The sinusoidal fluctuation with a single frequency and the flow fluctuation of the facility itself obtained by the SVD method are shown in Figure 15 and Figure 16. It could be found that the SVD method could extract the sinusoidal fluctuation signal with a single frequency and the flow fluctuation signal of the facility itself with eliminating noise at the same time. The experimental results of flow fluctuation are given in Table 7.

For experiment No. 2, the flow fluctuation calculated directly using the measured signal of the flowmeter was 1.01%, the flow fluctuation calculated using the signal obtained by the median filter was 0.95%, and it was 0.99% calculated using the signal obtained by the SVD method. In addition, using the extracted sinusoidal fluctuation signal with 1 Hz frequency and the flow fluctuation signal of the facility itself, the calculated fluctuations were 0.97% and 0.22%, respectively. For experiment No. 3, the flow fluctuation calculated directly using the measured signal of the flowmeter was 0.57%, the flow fluctuation calculated using the signal obtained by the median filter was 0.47%, and it was 0.53% calculated using the signal obtained by the SVD method. In addition, using the extracted sinusoidal fluctuation signal with an 8 Hz frequency and the flow fluctuation signal of the facility itself, the calculated fluctuations were 0.48% and 0.22%, respectively. For experiment Nos. 2 and 3, the flow fluctuation of the facility itself was consistent with the results of experiment No. 1.

When the flow fluctuation was calculated directly using the measured results of the flowmeter, the results contained a certain deviation. Through noise reduction by the SVD method, the flow fluctuation results were slightly smaller than direct calculation results because the noise effect in the measured results was eliminated. At the same time, the SVD method could accurately extract the single-frequency fluctuation signal from the superposition flow fluctuation signal of multiple frequencies. The results of the flow fluctuation calculated using the fluctuation signals obtained by the SVD method were very close to the real flow fluctuations, and the deviations in the flow fluctuation caused by noise were eliminated, which can be used to reflect the flow stability of the flow standard facility.

Although the result of the median filter was less than that of the direct calculation and even a little less than that of the proposed SVD method, the reliability of this result was not as good as that of the SVD method, because of its poor performance in amplitude attenuation and SNR improvement. Moreover, it was difficult to obtain the single-frequency fluctuation signal.

Through the experiment results, it could be found that the proposed SVD method in this paper could eliminate the superimposed noise in the flowmeter measured results and accurately obtain the real flow fluctuation signal from the noisy signal and superposition flow fluctuation signal of multiple frequencies, and then obtain accurate flow fluctuation of the flow standard facility, which could be used to reflect the stability of the flow standard facility.

## 6. Conclusions

In this paper, a new measuring method of flow fluctuation was proposed based on singular value decomposition (SVD), and data reconstruction was carried out to expand the single-dimensional data of the measured result to multi-dimensional data to realize the proposed SVD method. A simulation experiment on the fluctuation signal under different levels of noise was conducted, after the influences of the data selection window width and number of singular values on the measured results were analyzed. In addition, an experimental platform was set-up based on a water flow standard facility and a special designed flow fluctuation generator to conduct experiments on the output signal of a venturi flowmeter by the proposed SVD method.

Through the simulation and experiment results, it could be found that the measuring method proposed could effectively eliminate noise caused by the measuring flowmeter or measurement system and accurately obtain the flow fluctuation signal from the noisy signal or superposition flow fluctuation signal of multiple frequencies, to accurately measure the flow fluctuation of the flow standard facility, which is used to accurately evaluate the flow stability for improving the performance of the flow standard facility.

## Figures and Tables

**Table 1:** Amplitude attenuation and SNR improvement for 1 Hz signal.

**Noise**	Variance	0.01	0.1	1
	SNR	32.19 dB	9.16 dB	−13.86 dB
**SVD Method**	Attenuation	5.40 × 10^−3^ m^3^/h	2.52 × 10^−2^ m^3^/h	2.50 × 10^−2^ m^3^/h
	SNR	100.53 dB	77.26 dB	66.90 dB
**Median Filter**	Attenuation	5.61 × 10^−2^ m^3^/h	1.50 × 10^−1^ m^3^/h	4.99 × 10^−1^ m^3^/h
	SNR	47.62 dB	23.15 dB	−20.14 dB

**Table 2:** Amplitude attenuation and SNR improvement for 8 Hz signal.

**Noise**	Variance	0.01	0.1	1
	SNR	18.32 dB	−4.70 dB	−27.73 dB
**SVD Method**	Attenuation	5.41 × 10^−3^ m^3^/h	2.54 × 10^−2^ m^3^/h	2.87 × 10^−2^ m^3^/h
	SNR	86.42 dB	62.40 dB	48.49 dB
**Median Filter**	Attenuation	1.15 × 10^−2^ m^3^/h	2.49 × 10^−2^ m^3^/h	3.64 × 10^−2^ m^3^/h
	SNR	25.37 dB	3.52 dB	−21.06 dB

**Table 3:** Amplitude attenuation and SNR improvement for superposition signal.

**Noise**	Variance	0.01	0.1	1
	SNR	34.42 dB	11.39 dB	−11.63 dB
**SVD Method**	Attenuation	5.22 × 10^−3^ m^3^/h	−6.41 × 10^−3^ m^3^/h	7.66 × 10^−2^ m^3^/h
	SNR	119.18 dB	103.64 dB	46.56 dB
**Median Filter**	Attenuation	5.71 × 10^−2^ m^3^/h	1.58 × 10^−1^ m^3^/h	4.78 × 10^−1^ m^3^/h
	SNR	49.91 dB	26.79 dB	−9.51 dB

**Table 4:** Amplitude attenuation and SNR improvement for extracted signals.

**Noise**	Variance	0.01	0.1	1
	SNR	34.42 dB	11.39 dB	−11.63 dB
**1 Hz Signal**	Attenuation	−1.49 × 10^−3^ m^3^/h	1.85 × 10^−2^ m^3^/h	5.97 × 10^−2^ m^3^/h
	SNR	102.44 dB	69.03 dB	52.72 dB
**8 Hz Signal**	Attenuation	1.10 × 10^−3^ m^3^/h	3.15 × 10^−3^ m^3^/h	−1.45 × 10^−2^ m^3^/h
	SNR	96.16 dB	82.17 dB	36.27 dB

**Table 5:** Simulation results of 1 Hz and 8 Hz fluctuations.

**Signal**	Noise Variance	0	0.01	0.1	1
**1 HZ** **Signal**	Simulated fluctuation	1.00%	1.03%	1.22%	2.23%
	Median filter		1.00%	1.09%	1.64%
	SVD method		1.01%	1.03%	1.01%
**8 HZ** **Signal**	Simulated fluctuation	0.50%	0.54%	0.83%	2.04%
	Median filter		0.51%	0.67%	1.40%
	SVD method		0.51%	0.53%	0.51%

**Table 6:** Simulation result of superimposed fluctuation.

**Noise Variance**	0	0.01	0.1	1
**Simulated Fluctuation**	1.12%	1.14%	1.27%	2.28%
**Median Filter**		1.12%	1.17%	1.69%
**SVD Method**		1.12%	1.09%	1.06%
**1 Hz Signal**	1.00%	1.00%	0.97%	0.94%
**8 Hz Signal**	0.50%	0.50%	0.50%	0.49%

**Table 7:** Experimental results.

Experiment No.	1	2	3
Direct calculation	0.25%	1.01%	0.57%
Median filter	0.22%	0.95%	0.47%
SVD method	0.22%	0.99%	0.53%
Sinusoidal fluctuation	/	0.97%	0.48%
Facility fluctuation signal	/	0.22%	0.22%

## Data Availability

Not applicable.

## References

[1] Jaiswal S.K., Yadav S., Agarwal R. (2017). Design and development of a novel water flow measurement system. Measurement.

[2] Meng T., Fan S., Wang C., Shi H. (2018). Influence analysis of fluctuation parameters on flow stability based on uncertainty method. Rev. Sci. Instrum..

[3] Engel R., Baade H.J. (2015). Quantifying impacts on the measurement uncertainty in flow calibration arising from dynamic flow effects. Flow Meas. Instrum..

[4] Berrebi J., Deventer J.V., Delsing J. (2004). Reducing the flow measurement error caused by pulsations in flows. Flow Meas. Instrum..

[5] Meng T., Fan S., Wang C., Shi H., Li X. (2018). Flow stability evaluation method based on flow-pressure correlation. Flow Meas. Instrum..

[6] Reis M.N., Hanriot S. (2017). Incompressible pulsating flow for low Reynolds numbers in orifice plates. Flow Meas. Instrum..

[7] Blythman R., Persoons T., Jeffers N., Nolan K.P., Murray D.B. (2017). Localised dynamics of laminar pulsatile flow in a rectangular channel. Int. J. Heat Fluid Flow.

[8] International Organization for Standardization (2018). ISO/TR 3313: 2018. Measurement of Fluid Flow in Closed Conduits—Guidelines on the Effects of Flow Pulsations on Flow-Measurement Instruments.

[9] Berrebi J., Martinsson P.E., Willatzen M., Delsing J. (2004). Ultrasonic flow metering errors due to pulsating flow. Flow Meas. Instrum..

[10] Zhu Y. (2006). Study on the measurement error of differential pressure flowmeter in pulsating flow. Chin. J. Sci. Instrum..

[11] Gajan P., Mottram R.C., Hebrard P., Andriamihafy H., Platet B. (1992). The influence of pulsating flows on orifice plate flowmeters. Flow Meas. Instrum..

[12] International Organization for Standardization (1990). ISO 9368-1: 1990. Measurement of Liquid Flow in Closed Conduits by the Weighing Method-Procedures for Checking Installations—Part 1: Static Weighing Systems.

[13] Meng T., Shi H., Wang C., Wu B. (2020). Application of principal component analysis in measurement of flow fluctuation. Measurement.

[14] Guo H.X., Xie K. (2007). An improved method of adaptive median filter. J. Image Graph..

[15] Liu F., Zhang G.Q. (1996). Application of wavelet transform and FK algorithm in filtering. Oil Geophys. Prospect..

[16] Ke X., Xu J.W., Yang D. (1997). Application of wavelet transform in signal filtering. J. Univ. Sci. Technol. Beijing.

[17] Loizou P.C. (2007). Speech Enhancement: Theory and Practice.

[18] Dendrinos M., Bakamidis S., Carayannis G. (1991). Speech enhancement from noise: A regenerative approach. Speech Commun..

[19] De Moor B. (1993). The singular value decomposition and long and short spaces of noisy matrices. IEEE Trans. Signal Process..

[20] Sun L., Dang S., Zhang T., Liu Y. (2018). Experiment on the influence of flow stability on flowmeter measuring performance. J. Tianjin Univ..

[21] Li J.H., Su Y.X. (2008). Uncertainty of the commutator and stability measurement in liquid flowmeter standard system. Acta Metrol. Sin..

[22] China Institute of Metrology (2000). JJG 164. Liquid Flow Standard Device.

[23] Zhou X.Y., Gao C.W., Cao J.H. (2014). Principal component analysis method and its application in data noise reducing. Ordnance Ind. Autom..

[24] Duan H.M. (2004). Standard Device and Standard Meter Flow Standard Device for Liquid Flow.

